# A cost-effective innovation in anaemia management for paediatric patients with haemodialysis-dependent chronic kidney disease

**DOI:** 10.1007/s00467-025-06680-x

**Published:** 2025-03-01

**Authors:** Rebecca Preston, Demetria Theodorou, Kate Sinnott, Dean Wallace, Amrit Kaur

**Affiliations:** 1 Department of Paediatric Nephrology, Royal Manchester Children’s Hospital, Manchester University Hospitals NHS Foundation Trust, Oxford Road, Manchester, M13 9WL UK; 2 Wellcome Centre for Cell-Matrix Research, Division of Cell-Matrix Biology and Regenerative Medicine, School of Biological Sciences, Faculty of Biology Medicine and Health, Manchester Academic Health Science Centre, The University of Manchester, Manchester, M13 9PT UK

**Keywords:** Paediatric kidney failure, Iron deficiency anaemia, Haemodialysis, Ferric carboxymaltose, Intravenous iron therapy

## Abstract

**Background:**

Paediatric patients undergoing haemodialysis typically require intravenous (IV) iron therapy to replenish iron stores. Upon establishing our home haemodialysis service, the need for an efficient IV iron administration method prompted exploration beyond the conventional use of iron sucrose, which is associated with anaphylaxis and requires frequent infusions. Ferric carboxymaltose has a favourable safety profile and corrects iron deficiency with less frequent infusions. We aimed to establish if ferric carboxymaltose was a viable alternative in this patient group.

**Methods:**

This single-centre, uncontrolled retrospective cohort study assessed the effectiveness of ferric carboxymaltose in maintaining laboratory parameters (haemoglobin level, transferrin saturation and reticulocyte haemoglobin content) within target range in our home haemodialysis population. Secondly, we conducted a comparative analysis to establish maintenance efficacy of ferric carboxymaltose, versus iron sucrose over a 12-month period. Finally, we performed a cost-effectiveness analysis of IV iron therapy, considering cost per dose and per month of treatment.

**Results:**

Following ferric carboxymaltose infusion, we observed significant increases in haemoglobin level, transferrin saturation and reticulocyte haemoglobin content, which was maintained at 3-month post-infusion. Ferric carboxymaltose demonstrated comparable efficacy to iron sucrose in maintaining laboratory parameters. Strikingly, ferric carboxymaltose treatment was associated with significantly decreased number of infusions per month (~ tenfold) and a significant cost-saving (~ fivefold).

**Conclusions:**

This study underscores the clinical efficacy and economic benefits of ferric carboxymaltose as a viable treatment for iron deficiency anaemia in paediatric patients who are haemodialysis-dependent and highlights the potential for significant improvements in healthcare delivery, in terms of reducing frequency of hospital visits for this patient population.

**Graphical abstract:**

**Figure:**
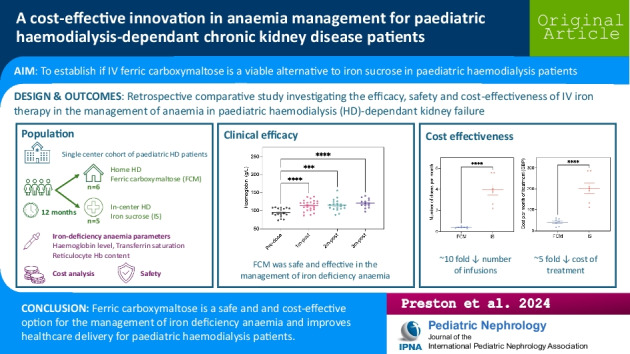
A higher resolution version of the Graphical abstract is available as Supplementary information

**Supplementary Information:**

The online version contains supplementary material available at 10.1007/s00467-025-06680-x.

## Introduction

Iron deficiency anaemia is a well-documented complication in children with chronic kidney disease (CKD) [1] and is associated with poor health outcomes and negative symptom reporting. Anaemia plays a critical role in reducing the quality of life for children suffering from CKD and contributes significantly to the progression of cardiovascular disease which represents a leading cause of mortality in children with kidney failure [2–5]. In this population, anaemia occurs secondary to both an absolute and functional iron deficiency. Absolute (true) iron deficiency is defined by severely reduced or absent iron stores, owing to the combination of reduced iron absorption and increased iron losses [6], whilst functional iron deficiency represents insufficient iron availability for incorporation into erythroid precursors [7].

Paediatric patients undergoing haemodialysis are at increased risk of iron deficiency due to the combination of gastrointestinal bleeding, frequent blood drawing and the haemodialysis process itself, and adequate iron stores are crucial to achieving optimal haemoglobin levels. Another important cause of anaemia in this population is the suboptimal production of erythropoietin, which stimulates red blood cell production. As kidney failure progresses, the decline in serum concentration of erythropoietin parallels kidney excretory functional loss, and erythropoietin deficiency becomes more pronounced [8]. The management of anaemia in CKD patients typically necessitates the administration of iron supplements and erythropoiesis stimulating agents, to replenish iron stores and support erythropoiesis. An inflammatory state induced by haemodialysis increases serum hepcidin levels which block iron absorption from the duodenum and iron release from stores in the liver and macrophages [9]. As such, oral iron therapy, which is often poorly tolerated by children, has limited efficacy in the management of anaemia in haemodialysis-dependent CKD patients.

At most tertiary paediatric nephrology centres, iron sucrose (IS) has long been the standard intravenous (IV) iron therapy for paediatric haemodialysis patients. IS necessitates administration of initial loading doses, followed by frequent maintenance infusions, often on a weekly basis. Although IS is effective in maintaining desirable haemoglobin levels (> 100 g/L), the frequent hospital visits required for its administration impose a significant burden on patients and their families, particularly those who travel long distances. More recently developed IV iron preparations, such as ferric carboxymaltose (FCM), allow correction of iron deficiency with less frequent infusions. FCM is a macromolecular hydroxide ferric carbohydrate complex designed for the controlled and gradual release of iron within cells, facilitated by uptake through endogenous iron-binding proteins [9]. The high stability of this complex ensures that ionic iron is not directly released into the bloodstream, thereby mitigating potential cellular damage from oxidative stress. This stability allows for the administration of substantial doses, up to 15 mg of iron per kilogramme via IV injection, whilst facilitating efficient delivery of iron to the reticuloendothelial system and subsequent distribution to the bone marrow, liver and spleen. The complex is metabolised in a manner that allows iron to be slowly and competitively transferred to endogenous iron-binding proteins [9].

Data from adult studies indicate that FCM is an effective alternative to IS and poses a more favourable safety profile [10–13]. Whilst IV iron therapy is associated with a small risk of minor hypersensitive reactions (~ 1:150–250) [14–17], severe adverse events are rare with the rate of anaphylaxis estimated to be less than 1 per 250,000 administrations [18]. In adult populations, several large multicentre studies have demonstrated a comparatively lower risk of hypersensitivity and anaphylaxis reactions with FCM, compared to other iron preparations [13, 14, 19]. Furthermore, a recent review has demonstrated the efficacy and promising safety profile of FCM in children and adolescents with iron delicacy anaemia of diverse aetiology [20]. However, no studies have investigated the efficacy or safety of FCM in the management of iron deficiency anaemia in paediatric patients with haemodialysis-dependent CKD.

To this end, we aimed to explore alternative iron preparations to reduce the frequency of hospital visits, and increase the safety of IV iron administration, for our paediatric home haemodialysis service in a UK tertiary paediatric nephrology centre. Using retrospective case-note review, we evaluated the efficacy, safety and cost-effectiveness of FCM versus IS in paediatric patients with haemodialysis-dependent CKD. As such, this study provides insights which will inform and enhance paediatric nephrology clinical practice nationwide.

## Methods

### Study design and participants

In this single-centre, uncontrolled, retrospective cohort study, we analysed the safety, efficacy and cost-effectiveness of IV iron therapy in the management of anaemia in children aged under 18 years with haemodialysis-dependent CKD in a UK-based tertiary paediatric nephrology centre. Children under the age of 18 years, undergoing in-centre or home haemodialysis, were included in the study. Patients with significant non-renal comorbidities were excluded. None of the patients included in this study were receiving concurrent oral iron treatment alongside IV iron therapy. To investigate the efficacy of FCM, we reviewed the case notes of children on home haemodialysis receiving FCM between the period of March 2020 until September 2023 (*n* = 6). To compare the efficacy of FCM to IS, we conducted a comparative retrospective case note review of home haemodialysis patients (FCM arm, *n* = 6) and in-centre haemodialysis patients (IS arm, *n* = 5) between the period of September 2022 until September 2023. To investigate the cost-effectiveness of FCM compared to IS, we conducted retrospective case note review on children receiving either FCM or IS (*n* = 7 per treatment group) between the period of March 2019 until September 2023 and compared number of doses of IV iron per month of haemodialysis and cost of IV iron per month of treatment (GBP). For the cost-effectiveness analysis, the number of patients studied per group increased as these individuals had received both IV iron preparations during the course of their treatment. Ethical approval was not required for this study as this was a retrospective study with anonymised patient data which aimed to improve patient care and service delivery.

### Iron preparations

As per the Children’s British National Formulary, this study used Ferinject® (as ferric carboxymaltose) and Venofer® (as iron sucrose) and dosing was calculated on mg/kg of body weight.

### Interpretation of laboratory values

Iron-deficiency anaemia in patients with CKD was defined by the National Institute of Clinical Excellence as haemoglobin level < 100 g/L, reticulocyte haemoglobin content < 29 pg and transferrin saturation < 20% [21] and target parameters for children receiving iron therapy are displayed in Table 1. To assess the efficacy of IV iron therapy, we collected data on haemoglobin level (g/L), transferrin saturation (%) and reticulocyte haemoglobin content (pg) from immediately prior to IV iron dosing (pre-dose) and at 1-month, 2-month and 3-month post-dose.

**Table 1:** Target parameters for children with CKD on iron therapy

Patient age	Haemoglobin level (g/L)	Transferrin saturation (%)	Reticulocyte haemoglobin (pg)
< 2 years	95–115	≥ 20	> 29
≥ 2 years	100–120	≥ 20	> 29

### Statistical analysis

Descriptive statistics were reported as means with 95% confidence intervals or standard deviation of the mean (for age). Differences in laboratory parameters before and after FCM were determined using an ordinary one-way ANOVA test for multiple comparisons. Differences in laboratory parameters in those receiving either FCM or IS were determined by Mann–Whitney *U* test and cost analysis between FCM and IS was determined by parametric unpaired two-tailed *t*-test. Data in figures are presented as mean ± SEM or median with interquartile range, as indicated in figure legends. Data were analysed following normality testing, as described in the legends and all data analysis was not blinded. Differences in means were considered statistically significant at *p* < 0.05. Significance levels are as follows: *p < 0.05; **p < 0.01; ***p < 0.001; ****p < 0.0001. Actual *p*-values are shown where appropriate. Statistical analyses were performed using the GraphPad Prism 9.5.1 software.

## Results

### Cohort characteristics

This study included children aged 18 years and under who were receiving IV iron therapy and undergoing home haemodialysis (FCM group) or in-centre haemodialysis (IS group) (Table 2). Mean age was 12.62 ± 5.35 in the FCM group and 11.99 ± 5.82 in the IS group. In the FCM group (*n* = 6), all study participants were male and were either of Asian ethnicity (50%) or White British (50%). In the IS group (*n* = 5), there was a slight male predominance (60%) and participants were of Asian ethnicity, except for one White British patient. In both groups, the underlying cause of kidney failure was varied, and all participants were receiving concurrent treatment with an erythropoietin stimulating agent (ESA).

**Table 2:** Summary of cohort characteristics

	Ferric carboxymaltose (FCM)	Iron sucrose (IS)
Gender		
Male	6 (100%)	3 (60%)
Female	-	2 (40%)
Age (years)		
< 6	2 (33%)	1 (20%)
6–12	1 (17%)	1 (20%)
13–18	3 (50%)	3 (60%)
Ethnicity		
White British	3 (50%)	1 (20%)
Asian	3 (50%)	4 (80%)
Concurrent ESA dose		
20 mcg weekly	2 (33%)	-
30 mcg weekly	1 (17%)	2 (40%)
40 mcg weekly	1 (17%)	1 (20%)
50 mcg weekly	1 (17%)	1 (20%)
> 50 mcg weekly	1 (17%)	1 (20%)
Primary kidney diagnosis		
Reflux nephropathy	2 (33%)	1 (20%)
Polycystic kidney disease (AR)	-	1 (20%)
Glomerular disease	2 (33%)	-
Syndromic	1 (17%)	2 (40%)
Unexplained (genetics negative)	1 (17%)	1 (20%)

### FCM is effective in the management of anaemia in paediatric patients with haemodialysis-dependent CKD

**Fig. 1:**
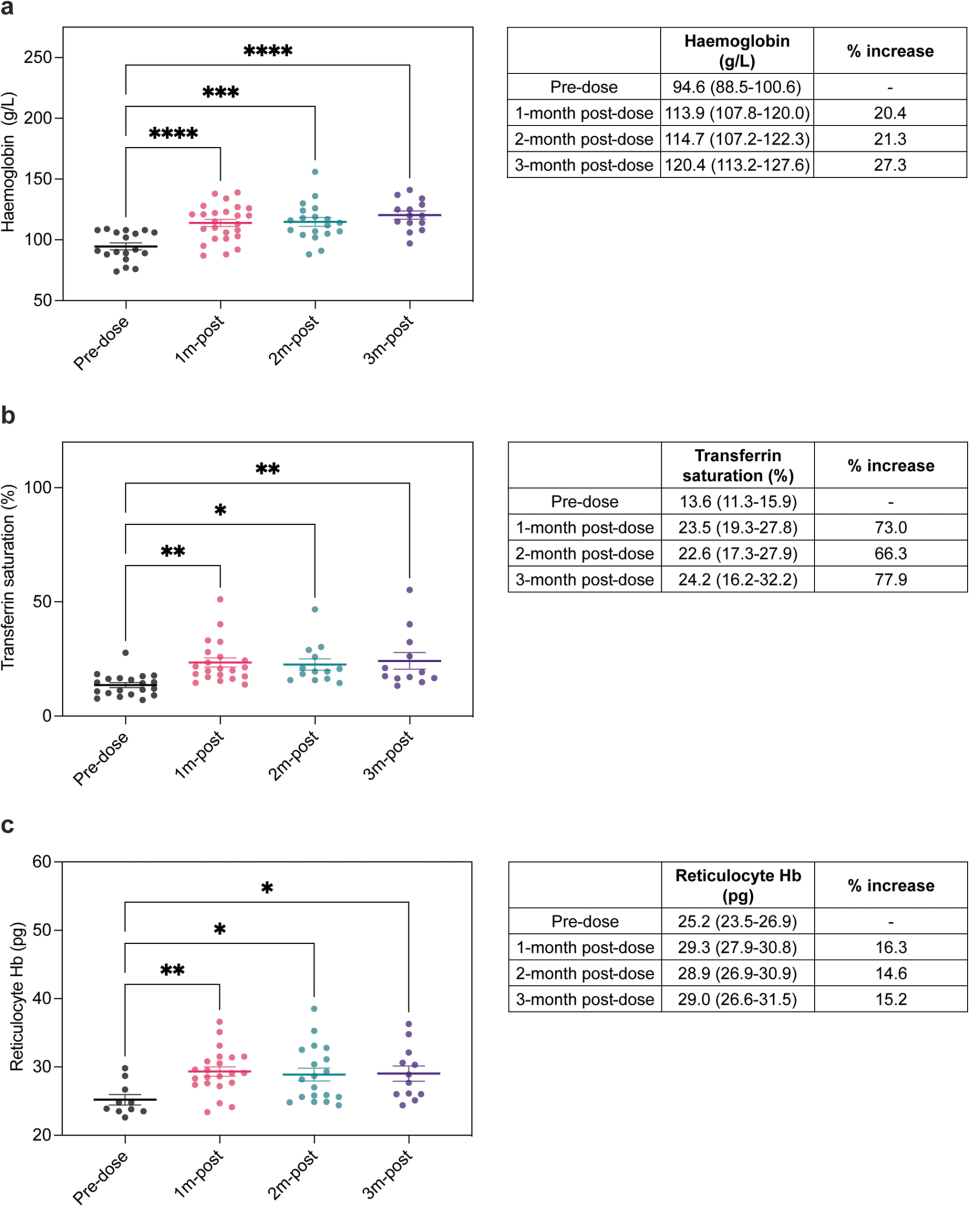
Efficacy of ferric carboxymaltose (FCM) in maintaining target parameters in paediatric haemodialysis-dependent CKD patients. **a** Haemoglobin level (g/L) immediately prior to FCM dosing and at 1-month, 2-month and 3-month post-administration (left panel). ****p = < 0.0001, ***p = 0.001, ****p = < 0.0001, by ordinary one-way ANOVA with multiple comparisons. Mean and 95% confidence interval of haemoglobin level (g/L) pre- and post-FCM and percentage increase in haemoglobin level (g/L) post-treatment (right panel). **b** Transferrin saturation (%) immediately prior to FCM dosing and at 1-month, 2-month and 3-month post-administration (left panel). **p = 0.0018, *p = 0.016, **p = 0.0048, by ordinary one-way ANOVA with multiple comparisons. Mean and 95% confidence interval of transferrin saturation (%) pre- and post-FCM and percentage increase in transferrin saturation (%) post-treatment (right panel). **c** Reticulocyte haemoglobin content (pg) immediately prior to FCM dosing and at 1-month, 2-month and 3-month post-administration (left panel). **p = 0.0087, *p = 0.0250, *p = 0.0354, by ordinary one-way ANOVA with multiple comparisons. Mean and 95% confidence interval of reticulocyte haemoglobin level (pg) pre- and post-FCM and percentage increase in reticulocyte haemoglobin level (pg) post-treatment (right panel). Bars on the graphs show mean ± SEM and coloured circles represent individual values. *n* = 6 patients receiving FCM. FCM, ferric carboxymaltose; IS, iron sucrose

In our home haemodialysis cohort (*n* = 6), FCM resulted in a significant increase in mean haemoglobin level at 1-month post-dose (113.9 g/L) and this was sustained after 2 months (114.7 g/L) and 3 months (120.4 g/L) of treatment compared to pre-dose levels (94.6 g/L) (Fig. 1a). Similarly, significant increases in transferrin saturation (%) and reticulocyte haemoglobin content (pg) were demonstrated at 1-month, 2-month and 3-month post-FCM therapy when compared to pre-dose levels (Fig. 1b and c). Notably, prior to FCM dosing, mean haemoglobin (94.6 g/L), transferrin saturation (13.6%) and reticulocyte haemoglobin levels (25.2 pg) were suboptimal but a single dose of FCM brought these parameters within the target range for children with CKD receiving iron therapy, as defined by the National Institute of Clinical Excellence (Table 1). Importantly, these parameters were maintained within target range at 3-month post-treatment. Indeed, at 3-month post-FCM therapy, mean haemoglobin levels were increased by 27.3%, transferrin saturation by 77.9% and reticulocyte haemoglobin content by 15.2%, compared to pre-dose levels. Within our patient cohort, no adverse events were noted in patients receiving FCM or IS therapy. Our data demonstrates that FCM is both a safe and effective treatment for iron-deficiency anaemia in paediatric patients with haemodialysis-dependent CKD.

### FCM is a cost-effective alternative to IS in the management of anaemia in paediatric patients with haemodialysis-dependent CKD

**Fig. 2:**
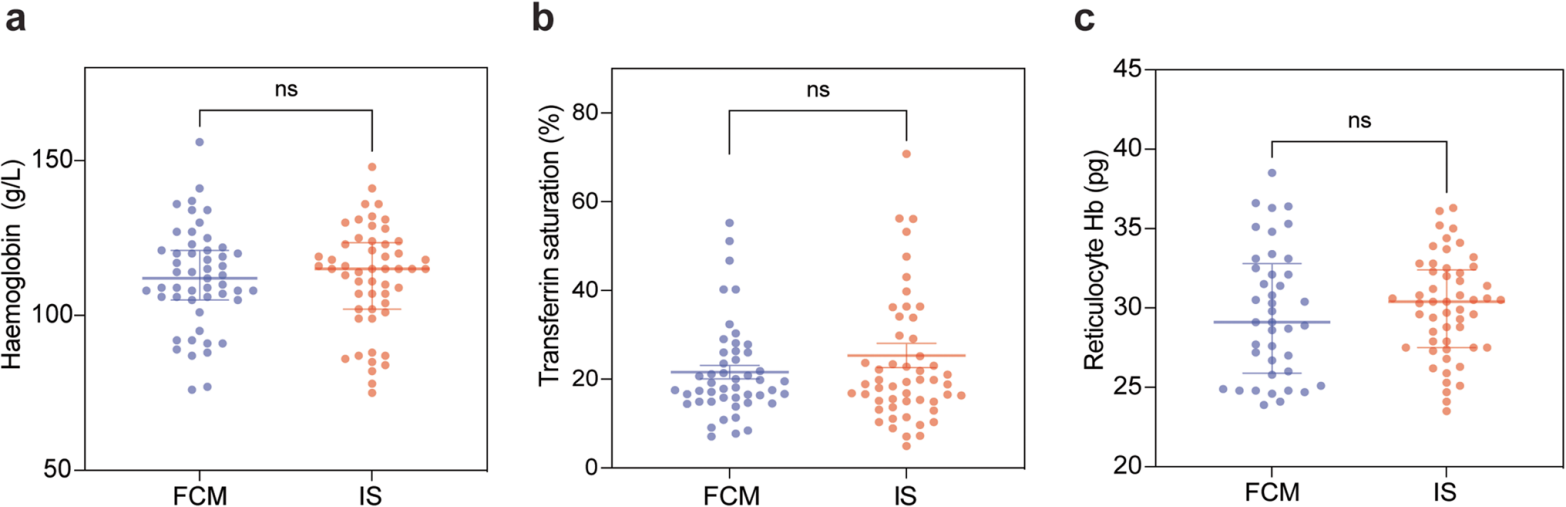
Comparative analysis of iron-deficiency anaemia laboratory values in patients receiving ferric carboxymaltose (FCM) versus iron sucrose (IS). **a** Haemoglobin levels (g/L), **b** transferrin saturation (%) and **c** reticulocyte haemoglobin content (pg) in patients receiving FCM (*n* = 6) versus IS (*n* = 5) over a 12-month period. Non-significant differences were determined by Mann–Whitney *U* test. Bars show median with interquartile range and coloured circles represent individual values. FCM, ferric carboxymaltose; IS, iron sucrose

**Fig. 3:**
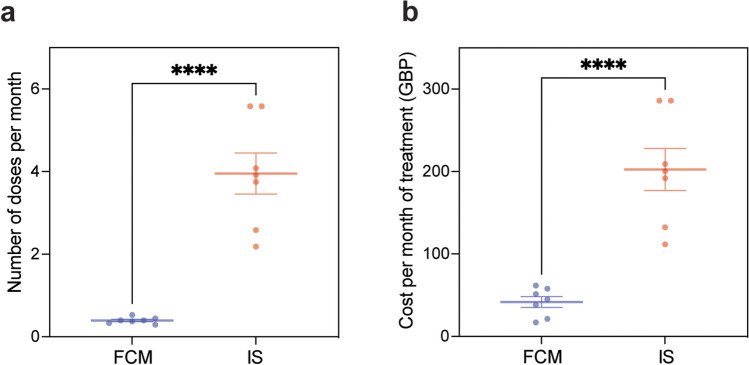
Cost-analysis of ferric carboxymaltose (FCM) versus iron sucrose (IS) in the management of anaemia in paediatric haemodialysis-dependent CKD patients. **a** The number of doses of FCM per month required to maintain target parameters within range was significantly less than IS. ****p = < 0.0001 by parametric unpaired two-tailed *t*-test. **b** The cost of FCM per month of treatment is significantly less compared to IS. ****p = < 0.0001 by parametric unpaired two-tailed *t*-test. Bars show mean ± SEM (*n* = 7 per treatment group). Coloured circles represent **a** mean number of doses per month and **b** mean cost of treatment per month for individual patients. FCM, ferric carboxymaltose; IS, iron sucrose

In our tertiary paediatric nephrology department, IS was the IV iron preparation of choice for patients receiving in-centre haemodialysis whilst our home haemodialysis cohort, received FCM. For in-centre haemodialysis patients, frequent IV infusions were required to maintain parameters within target range (Table 1), and this was associated with a significant financial burden. To investigate the efficacy of FCM versus IS, we performed a comparative analysis of iron-deficiency anaemia laboratory values in patients receiving in-centre haemodialysis, and therefore IS (*n* = 5), and patients on home haemodialysis who received FCM (*n* = 6), over a 12-month period. We detected no significant difference in haemoglobin levels, transferrin saturation or reticulocyte haemoglobin content between the two groups (Fig. 2). Indeed, median haemoglobin levels were 112 g/L (FCM group) compared to 115 g/L (IS group) (Fig. 2a); median transferrin saturations were 21.6% (FCM group) compared to 25.3% (IS group) (Fig. 2b); and median reticulocyte haemoglobin levels were 29.1 pg (FCM group) compared to 30.4 pg (IS group) (Fig. 2c). Having established comparable efficacy between FCM and IS in maintaining laboratory parameters within target range, we next performed a cost analysis to determine if FCM would offer the dual benefit of reducing the number of IV infusions required to maintain target parameters, whilst alleviating the financial burden of IV iron therapy in this patient group. We compared the number of doses received by patients on FCM (*n* = 7) compared to those on IS (*n* = 7) from conception of our home haemodialysis service in March 2019 until September 2023. Strikingly, in-centre haemodialysis patients on IS received ten times more IV iron infusions with on average, four IV infusions being administered every month in order to maintain parameters within target range (Fig. 3a). Comparatively, our home haemodialysis patients receiving FCM required just one IV iron infusion every 2.5 months to maintain target parameters (Fig. 3a). Next, we calculated the cost per month (GBP; £) based on prescriptions of IV iron per infusion, using the NHS indicative price as stated in the Children’s British National Formulary [22] (Table 3). We demonstrate that the average cost of IS per month was significantly more expensive than FCM (Fig. 3b). Indeed, the average cost of IS per haemodialysis patient was £202.47 (GBP) per month of treatment compared to just £41.79 (GBP) for FCM. Importantly, this fivefold cost-saving could be further amplified with careful prescribing practices, paying attention to the dose required (mg/kg) for individual patients and selecting the appropriate formulation. Overall, our data demonstrates that FCM is as effective in maintaining laboratory parameters within target range, compared to IS, in the management of anaemia in paediatric patients with haemodialysis-dependent CKD, whilst offering the dual advantage of significantly decreased number of infusions per month (~ tenfold) and a significant cost-saving (~ fivefold).

**Table 3:** Indicative price of medicinal forms of iron sucrose and ferric carboxymaltose as per the Children’s British National Formulary

Drug	Available solutions	Amount	Cost (GBP)	Cost per vial (GBP)	Cost per mg (GBP)
IV Venofer® (iron sucrose)	100 mg/5 mL	1 vial	£51.20	£51.20	£0.51
IV Ferinject® (ferric carboxymaltose)	1000 mg/20 mL	1 vial	£154.23	£154.23	£0.15
	500 mg/10 mL	5 vials	£477.50	£95.50	£0.19
	100 mg/2 mL	5 vials	£95.50	£19.10	£0.19

## Discussion

In the UK, FCM is licensed for use in children over 14 years of age. Although not formally approved for younger children, FCM has been used off-label, safely and effectively in paediatric patients aged 9 months to 18 years with iron-deficiency anaemia [23]. Elsewhere in Europe and in the USA, FCM is approved for use in children aged 1 year and older for the treatment of iron deficiency anaemia when oral iron preparations are contraindicated, ineffective or poorly tolerated [24, 25]. FCM has been shown to be effective in the management of anaemia in adult populations and poses a favourable safety profile to other IV iron preparations, with comparatively fewer hypersensitivity and anaphylaxis episodes [13, 14, 19]. Our study is the first to report on the use of FCM for the management of iron deficiency anaemia in a paediatric haemodialysis cohort, demonstrating its efficacy and practicality in this specific patient population.

### FCM in children

To date, no randomised controlled trials or direct comparative studies have been conducted on the use of FCM in children. A recent and comprehensive review by Aksan et al. evaluated 33 publications detailing efficacy data from case reports and retrospective studies reporting the use of FCM in paediatric populations [20]. This review concluded that FCM is effective in children under 14 years, demonstrating significant improvements in haemoglobin, iron and ferritin levels. Safety data was analysed from 25 studies and was consistent with the known safety profile for FCM. Notably, several studies involving a cumulative total of 93 children reported no adverse events.

### FCM in non-haemodialysis-dependent CKD

In 2014, the FIND-CKD study evaluated the efficacy and safety of FCM, compared to oral iron therapy, in 626 adult patients with iron deficiency anaemia secondary to non-dialysis-dependent CKD [12]. This multi-centre, open-label, randomised trial demonstrated that FCM achieved greater increases in haemoglobin levels compared to oral iron therapy, as well as more effectively maintaining target ferritin and transferrin saturation. Furthermore, FCM was generally well-tolerated and demonstrated a favourable safety profile, with no evidence of increased renal toxicity, cardiovascular disease or infectious events. Since then, the safety and effectiveness of FCM has been investigated in paediatric patients with CKD non-dependent on haemodialysis [9]. This retrospective study included 79 patients, with a mean age of 9 years. The authors nicely demonstrate efficacy data for FCM with a single infusion resulting in a median increase of 1.4 g/dL in haemoglobin, 224 µg/L in ferritin, 37 µg/dL in serum iron and 18% in transferrin saturation, after 15–45 days. Again, FCM was well tolerated, with no serious adverse events. As such, this study concluded that FCM is a safe and effective treatment for iron deficiency anaemia in paediatric patients with non-dialysis-dependent CKD.

### FCM in haemodialysis

There are few studies investigating the safety and efficacy of FCM in the management of anaemia in patients with haemodialysis-dependent CKD. In the largest study to date, this was evaluated in 150 adult patients with iron deficiency anaemia undergoing haemodialysis [11]. Here, FCM successfully increased haemoglobin levels from 9.1 to 10.3 g/dL, with 61.7% of patients responding to treatment, and attaining ≥ 1.0 g/dL increase in haemoglobin level from baseline. In terms of safety, FCM was well-tolerated with just 3.1% of patients discontinuing treatment due to adverse events. A second prospective observational study, including 31 adult haemodialysis patients [26], demonstrated that maintenance dosing of FCM was effective in sustaining ferritin levels for up to 3 weeks, whilst transferrin saturation and haemoglobin levels did not change significantly from baseline. Unfortunately, this study did not report on safety outcomes. Taken together, these limited findings suggest that FCM is a safe and effective therapy in the management of iron deficiency anaemia in patients with haemodialysis-dependent CKD. However, the absence of studies investigating the clinical utility of FCM in paediatric haemodialysis patients represents a significant short-falling and highlights the need for further studies in this population.

### FCM in paediatric patients with haemodialysis-dependent CKD

In our single-centre, retrospective, cohort study, we demonstrate significant increases in mean haemoglobin level, transferrin saturation and reticulocyte haemoglobin content at 1 month, 2 months and 3 months after FCM dosing, compared to baseline levels. There were no adverse events reported from patients receiving either FCM or IS therapy. This suggests that FCM is a safe and effective treatment for managing iron deficiency anaemia in paediatric haemodialysis patients. Importantly, FCM demonstrated comparative efficacy to IS, which until now represents the gold standard IV iron therapy in paediatric patients with CKD.

FCM dosing poses a key advantage over that of IS, with less frequent and shorter duration infusions required to reach desirable haemoglobin levels. Indeed, in our patient cohort, we demonstrate that target haemoglobin levels were maintained with 10 times less frequent IV iron infusions with FCM, compared to IS. The streamlined administration schedule for FCM significantly reduces the burden of frequent hospital visits for home haemodialysis patients, enhancing compliance and convenience. Additionally, the reduced frequency of infusions required with FCM treatment translates to lower nursing time and resource utilisation, benefiting both patients and healthcare providers. Our cost analysis further supports the implementation of FCM as a cost-effective alternative to IS in the UK. By evaluating the cost of FCM per dose and per month of therapy, we demonstrate an approximate fivefold cost-saving with FCM which could be further amplified by collaboration with pharmacy colleagues to ensure optimal prescribing strategies. In turn, this significant economic advantage enables home haemodialysis programmes to allocate resources more efficiently.

Overall, our study underscores the clinical efficacy and economic benefits of FCM as a viable treatment for iron deficiency anaemia in paediatric patients with haemodialysis-dependent CKD and highlights the potential for significant improvements in healthcare delivery, in terms of reducing the frequency of hospital visits, for this patient population. Future research efforts should focus on confirming these findings in larger cohorts of paediatric patients on haemodialysis.

### Limitations

A key limitation of this study is its retrospective design which inherently restricts the ability to establish causality and increases susceptibility to selection bias. Furthermore, our study evaluated the use of FCM which was only received at our tertiary centre by home haemodialysis patients, thus restricting our study participants significantly. Our small sample size limits the generalisability of our findings to the broader paediatric haemodialysis population. Having now adopted FCM as the IV iron therapy of choice for our in-centre haemodialysis patients, we will be in a position to provide updated data on a larger scale in the near future. Secondly, our study made no adjustment for confounding factors such as nutritional status, degree of secondary hyperparathyroidism, dialysis efficacy or compliance to other medication which may have been optimised in our home haemodialysis cohort compared to our in-centre haemodialysis cohort. Finally, as this was a UK-based study with cost-analysis projections based on NHS indicative pricing, worldwide cost-savings may not be comparable. Despite these limitations, our study provides a valuable starting point given the paucity of reported studies on the use of FCM in this specific patient group. Future prospective studies with larger cohorts are necessary to validate these preliminary findings and to better understand the long-term safety and efficacy of FCM in paediatric haemodialysis patients.

## Supplementary Information

Below is the link to the electronic supplementary material.

Graphical Abstract (PDF 202 KB)

## Data Availability

The datasets generated and analysed during the current study are available from the corresponding author on reasonable request.
