# Supplementary material for: A cost-effective innovation in anaemia management for paediatric patients with haemodialysis-dependent chronic kidney disease
Source: Pediatr Nephrol. 2025 Mar 1;40(7):2353–61. doi: 10.1007/s00467-025-06680-x (PMC12116716; doi:10.1007/s00467-025-06680-x)
Supplement: Supplementary file 1 — Graphical Abstract (PDF 202 KB) [file 467_2025_6680_MOESM1_ESM.pdf]

A cost-effective innovation in anaemia management for paediatric haemodialysis-dependant chronic kidney disease patients

Original Article

AIM: To establish if IV ferric carboxymaltose is a viable alternative to iron sucrose in paediatric haemodialysis patients

DESIGN & OUTCOMES: Retrospective comparative study investigating the efficacy, safety and cost-effectiveness of IV iron therapy in the management of anaemia in paediatric haemodialysis (HD)-dependant kidney failure

Population

Single center cohort of paediatric HD patients

Home HD
Ferric carboxymaltose (FCM)
n=6

12 months

In-center HD
Iron sucrose (IS)
n=5

Iron-deficiency anaemia parameters
Haemoglobin level, Transferrin saturation
Reticulocyte Hb content

Cost analysis

Safety

Clinical efficacy

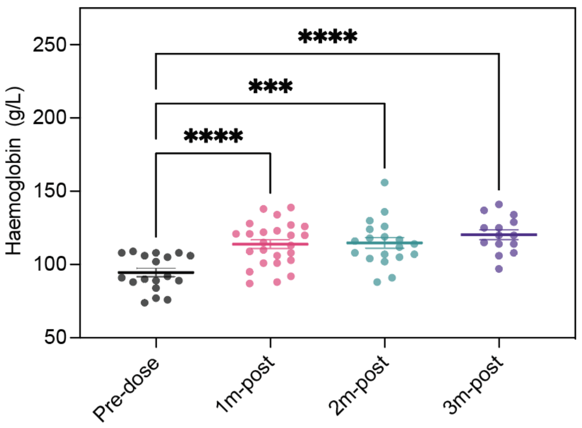

FCM was safe and effective in the management of iron deficiency anaemia

Cost effectiveness

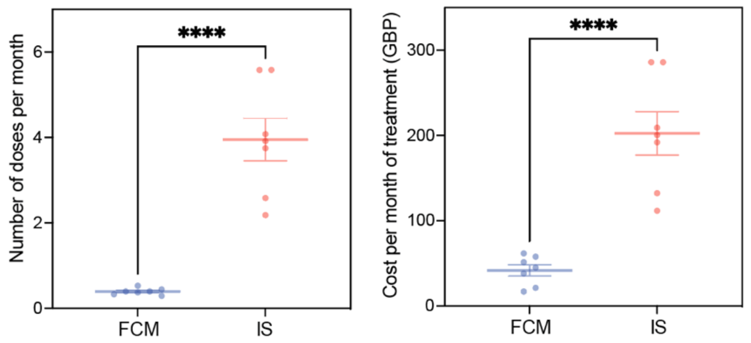

~10 fold ↓ number of infusions

~5 fold ↓ cost of treatment

CONCLUSION: Ferric carboxymaltose is a safe and and cost-effective option for the management of iron deficiency anaemia and improves healthcare delivery for paediatric haemodialysis patients.

Preston et al. 2024

Pediatric Nephrology
Journal of the
International Pediatric Nephrology Association
IPNA
